# Comparison of Zeiss MEL90 and Alcon WaveLight EX500 Excimer Lasers in FDA Premarket Approval Trials for the Treatment of Myopia, Hyperopia, and Mixed Astigmatism

**DOI:** 10.3390/jcm14155403

**Published:** 2025-07-31

**Authors:** Traeson M. Brandenburg, Mina M. Sitto, Phillip C. Hoopes, Majid Moshirfar

**Affiliations:** 1 Hoopes Vision Research Center, Hoopes Vision, Draper, UT 84020, USA; 2 Creighton University School of Medicine, Omaha, NE 68178, USA; 3 Department of Ophthalmology, Wayne State University School of Medicine, Detroit, MI 48201, USA; 4 John A. Moran Eye Center, University of Utah School of Medicine, Salt Lake City, UT 84123, USA; 5 Utah Lions Eye Bank, Murray, UT 84107, USA

**Keywords:** LASIK, WaveLight, EX500, MEL90, refractive surgery, safety, predictability, excimer laser

## Abstract

**Background/Objectives**: Although both the MEL90 (Carl Zeiss Meditec AG, Jena, Germany) and WaveLight EX500 (Alcon Laboratories, Inc., Fort Worth, TX, USA) are two widely used excimer lasers, comparisons between the two remain limited. This study evaluates visual and refractive outcomes from the U.S. Food and Drug Administration (FDA) premarket approval trials of these platforms in the treatment of myopia with and without astigmatism, hyperopia with and without astigmatism, and mixed astigmatism. **Methods**: Clinical outcomes from FDA premarket approval trials were compared between the recently approved MEL90 and the WaveLight (now termed EX500) excimer lasers. **Results**: A total of 714 eyes (358 patients) from MEL90 and 1353 eyes (706 patients) from EX500 were analyzed up to 6 months postoperatively. In the hyperopia/hyperopic astigmatism cohort, the EX500 demonstrated greater efficacy relative to MEL90, with more eyes achieving a postoperative uncorrected distance visual acuity (UDVA) of 20/20 or better (48.6% vs. 68.7%, respectively; *p* < 0.001). In both the MEL90 and EX500, at least 85% of eyes with myopia/myopic astigmatism and 68% with mixed astigmatism achieved a postoperative UDVA of 20/20 or better. For all refractive cohorts, more than 95% of eyes achieved a UDVA of 20/40 or better at 6 months (all *p* > 0.05). The EX500 was more likely to demonstrate an improvement of more than two lines of UDVA compared to baseline CDVA (all *p* < 0.05). In contrast, the MEL90 showed greater predictability of spherical equivalent within ±0.50 D and ±1.00 D for the hyperopia/hyperopic astigmatism cohort (both *p* = 0.007), as well as within ±0.50 D for the myopia/myopic astigmatism cohort (*p* < 0.001). Postoperatively, both platforms were associated with decreased glare and halos, although findings were variable in the EX500 mixed astigmatism cohort. **Conclusions**: Both excimer lasers demonstrated safe and effective outcomes that exceed the threshold set by the FDA.

## 1. Introduction

As of 2023, vision impairment due to refractive error affects an estimated 2.2 billion individuals worldwide [1]. Specifically, myopia, hyperopia, and astigmatism have estimated prevalences of 26.5%, 30.9%, and 40.4%, respectively [2]. Myopia, in particular, is projected to impact at least 50% of the global population by 2050 [3]. Advancements in refractive surgery continue to evolve in parallel with this growing demand [4]. Since the patent of laser-assisted in situ keratomileusis (LASIK) in 1989 by Dr. Gholam Peryman, the procedure has consistently shown safety and efficacy in the treatment of myopia and myopic astigmatism, hyperopia and hyperopic astigmatism, and mixed astigmatism [5]. Although multiple techniques have been developed for reshaping the cornea, the excimer laser used in LASIK remains the gold standard [5]. The most recent Food and Drug Administration (FDA)-approved excimer laser in the United States is the MEL90 (Carl Zeiss Meditec AG, Jena, Germany), which was approved on 4 December 2024 [6].

The MEL90 was first showcased at the European Society of Cataract and Refractive Surgeons Annual Meeting in 2013 as Zeiss’ successor to the MEL80. Since that time, the MEL90 has been widely adopted internationally, but lacked U.S. FDA approval until recently. Conversely, the WaveLight excimer laser series (Alcon Laboratories, Inc., Fort Worth, TX, USA) has been well-established in the U.S. since its approval in 2003 [7]. There are limited comparisons available between the clinical outcomes of MEL90 and those of other excimer lasers, given its recent approval. This study evaluates the FDA premarket approval (PMA) visual outcomes of the MEL90 with Alcon’s WaveLight (now known as the EX500), as well as comparing these findings to those of large cohort published studies on the treatment of myopia with and without astigmatism, hyperopia with and without astigmatism, and mixed astigmatism.

## 2. Materials and Methods

A retrospective analysis of the PMA trials for MEL90 and WaveLight excimer lasers was conducted using the publicly available U.S. FDA Summary of Safety and Effectiveness Data (SSED). The MEL90 PMA data spanned from 2019 to 2023 and includes clinical outcomes for the treatment of myopia with and without astigmatism, hyperopia with and without astigmatism, and mixed astigmatism. In contrast, the original WaveLight PMA trials were conducted from 2001 to 2002 for the treatment of myopia or hyperopia with and without astigmatism, and from 2004 to 2005 for mixed astigmatism.

### 2.1. FDA Approval Timeline

The MEL80 excimer laser (P060004) by Zeiss received initial FDA approval on 11 August 2006, for the correction of myopia up to −7.00 D of sphere and −3.00 D of cylinder. This was expanded under supplement S001 on 28 March 2011, to include the correction for hyperopia up to 5.00 D of sphere and 3.00 D of cylinder. The MEL90 received expanded approval (S006) on 4 December 2024, for correcting myopia up to −10.00 D sphere and up to −4.00 D cylinder, hyperopia up to 4.00 D sphere and 3.00 D cylinder, and mixed astigmatism with cylinder up to 4.00 D [6].

Alcon initially received approval for the WaveLight ALLEGRETTO WAVE Excimer Laser System (P020050) on 7 October 2003, for the correction of myopia up to −12.00 D of sphere and up to −6.00 D of cylinder. This was followed shortly by a separate PMA (P030008) on 10 October 2003, to include the correction of hyperopia up to 6.00 D of sphere and 5.00 D of cylinder. A subsequent expansion (P030008/S004) was approved on 9 April 2006, to include the treatment of mixed astigmatism up to 6.00 D. The ALLEGRETTO WAVE EYE-Q Excimer Laser System was approved in 2007 (P020050/S003), followed by the WaveLight EX500 Excimer Laser System in 2010 (P020050/S006), which was approved under the original product code for the ALLEGRETTO WAVE with a new supplemental designation [7,8,9]. This paper will refer to the original WaveLight ALLEGRETTO WAVE Excimer Laser platform as the EX500.

It is important to note that the EX500 also received FDA approval for performing wavefront-guided LASIK when used in conjunction with the WaveLight Analyzer (26 July 2006; P020050/S004), topography-guided LASIK with the ALLEGRO Topolyzer (27 September 2013; P020050/S012), and photorefractive keratectomy (16 November 2016; P020050/S023). However, these refractive procedures are excluded from the present study to allow direct comparison with the MEL90 [10,11,12].

### 2.2. Laser Specifications

The MEL80 operates at a frequency of 250 Hz. The newest model, the MEL90, incorporates a FLEXIQUENCE switch function that operates at either 250 or 500 Hz. The ablation profiles of these excimer lasers are comparable. The MEL90 operates at a wavelength of 193 nm and features an ablation profile of up to 9.2 mm, comprising an optical zone of 6.0–7.0 mm and a transition zone of 2.2 mm. MEL90 has an ablation rate of 500 Hz and an active eye-tracker frequency of 1050 Hz, providing a latency of 2 milliseconds.

The ALLEGRETTO WAVE Excimer Laser System operates at 200 Hz, while the ALLEGRETTO WAVE EYE-Q Excimer Laser System operates at a greater frequency of 400 Hz. The newest model, the WaveLight EX500, operates at 500 Hz and shares the same ablation profiles as its predecessors. Similar to the MEL90, the EX500 operates at 193 nm but has an ablation diameter of up to 9.0 mm, with an optical zone of 6.0 or 6.5 mm and a transition zone of 2.5–3.0 mm. It also incorporates an eye tracker with a rate of 1050 Hz and a 2-millisecond latency (Table 1).

### 2.3. PMA Cohorts

The MEL90 PMA trials enrolled 836 eyes from 418 subjects. Following screening, 358 eyes (183 subjects) were included for the treatment of myopia or myopic astigmatism, 221 eyes (117 subjects) for hyperopia or hyperopic astigmatism, and 135 eyes (77 subjects) for mixed astigmatism. There were 19 subjects with left and right eyes assigned to different cohorts (8 were assigned to both myopia and mixed astigmatism, and 11 to hyperopia and mixed astigmatism). The EX500 PMA trials included 901 eyes treated for myopia or myopic astigmatism, 290 eyes for hyperopia or hyperopic astigmatism, and 162 eyes for mixed astigmatism. The three cohorts are hereafter referred to as the myopic cohort, the hyperopic cohort, and the mixed astigmatism cohort.

All patients within the PMA clinical trials were followed at 1 day, 1 week, 1 month, 3 months, 6 months, 9 months, and 12 months. The present study compares both MEL90 and EX500 up to 6 months due to incomplete or uneven reporting across all groups. The follow-up at 6 months for MEL90 was 95.8% (684/714 eyes). In the EX500 study, follow-up rates at 6 months were 91.9% (818/901 eyes) for the myopic cohort, 90.3% (151/290 eyes) for the hyperopic cohort, and 68.5% (111/162 eyes) for the mixed astigmatism cohort. Parameters were only included when available for both platforms in the FDA PMA data. All available correction indices stratified by preoperative cylinder were included despite varying follow-up durations, due to their clinical relevance.

### 2.4. Outcome Measures

Five primary outcomes were assessed: (1) efficacy, measured by postoperative uncorrected distance visual acuity (UDVA) and the number of lines better than preoperative best-corrected distance visual acuity (CDVA) compared to postoperative UDVA; (2) safety, evaluated by preoperative CDVA compared to postoperative CDVA; (3) accuracy, based on postoperative manifest refraction spherical equivalent (MRSE); (4) patient-reported outcomes; and (5) complication rates.

### 2.5. Selection of Published Studies

A literature search was conducted to identify published literature reporting visual outcomes for the MEL90 or EX500 platforms. To find relevant studies, PubMed and Google Scholar were searched using various combinations of keywords: “MEL90”, “EX500”, “LASIK”, “WaveLight”, “Allegretto”, “myopia”, “mixed astigmatism”, “hyperopia”, and “retrospective”. Studies were selected if they reported visual outcomes, had a sample size of at least 50 eyes, and had at least 3 months of follow-up. Prospective studies were included irrespective of sample size.

### 2.6. Statistical Analysis

Statistical analyses were conducted using SPSS (version 30.0; IBM Corporation, Armonk, NY, USA) and Python (version 3.13; Python Software Foundation) with the SciPy (1.15), NumPy (version 2.2), and Pandas (version 2.2) libraries. Welch’s *t*-test was used to compare continuous variables in demographic data, which accounts for both unequal variance and unequal, yet sufficiently large, sample sizes. Categorical variables, such as safety, efficacy, and MRSE accuracy, were assessed using Pearson’s chi-square test and Fisher’s exact test when appropriate. A *p*-value of less than 0.05 was considered statistically significant.

Due to the absence of raw data, normality tests such as the Shapiro–Wilk test and the Kolmogorov–Smirnov test could not be performed. Similarly, adjustments for preoperative differences using covariates were not feasible. However, normality of the sampling distribution was assumed based on the large sample sizes. The generation of nine standard graphs and astigmatic analysis by Alpins method was also not performed for this reason. The MEL90 reported outcomes separately for the myopia, hyperopia, and mixed astigmatism cohorts at 6 months, without reporting aggregated data across all cohorts. Therefore, all comparisons between platforms, except for astigmatic accuracy, were limited to this time point. For the MEL90, MRSE was calculated using the overall mean values of sphere and cylinder. For the EX500, only refractive bins for MRSE were available. Therefore, the midpoint of each bin was used to approximate MRSE, and a weighted average was calculated based on the number of eyes per bin. Standard deviations for the stratified correction indices of EX500 were not reported; therefore, this statistical analysis could not be performed. Patient-reported visual outcomes could also not be statistically compared due to differences in questionnaire parameters and time points between platforms.

## 3. Results

### 3.1. Patient Demographics

Across all three cohorts, the EX500 platform was associated with a significantly older mean age (all *p* < 0.001) (Table 2). In the myopia cohort, the MEL90 demonstrated more myopic preoperative MRSE compared to the EX500 (*p* < 0.001). This cohort, as well as the hyperopia cohort, also had significantly greater baseline cylinder (*p* = 0.017 and <0.001, respectively). All other baseline refractive parameters were comparable between platforms.

### 3.2. Efficacy

**Figure 1:**
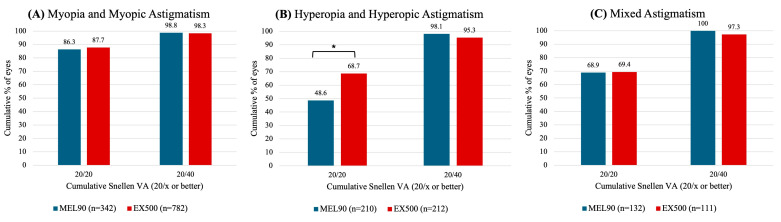
Comparison of uncorrected distance visual acuity (UDVA) between MEL90 and EX500 (**A**) myopia and myopic astigmatism, (**B**) hyperopia and hyperopic astigmatism, and (**C**) mixed astigmatism at 6 months. * Indicates statistically significant (*p* < 0.05).

At 6 months, over 85% of eyes in both myopia cohorts achieved a UDVA of 20/20 or better (*p* = 0.497) (Figure 1A). The EX500 hyperopia cohort was significantly more likely to achieve an UDVA of 20/20 or better compared to those treated with MEL90 (*p* < 0.001) (Figure 1B). In the mixed astigmatism cohort, over 68% of eyes in both platforms achieved an UDVA of 20/20 or better (*p* = 0.942) (Figure 1C). Across all cohorts, more than 95% of all eyes achieved a postoperative UDVA of 20/40 or better (*p* = 0.533) (Figure 1A–C).

#### 3.2.1. Myopia with and Without Astigmatism

**Figure 2:**
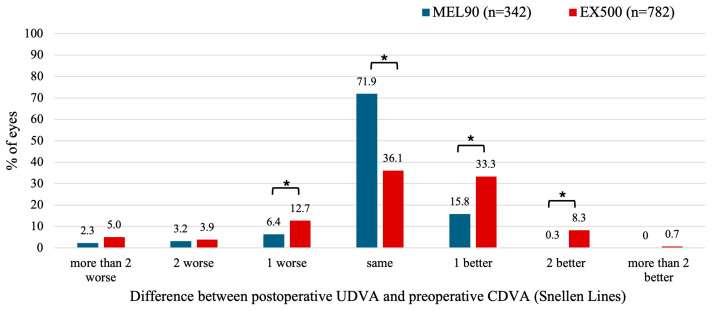
Preoperative corrected distance visual acuity (CDVA) versus uncorrected distance visual acuity (UDVA) for the myopia cohort at 6 months. LogMAR values from the MEL90 studies were converted to Snellen equivalents for comparison. * Indicates statistically significant (*p* < 0.05).

For the EX500, 12.7% of eyes showed a postoperative UDVA that was one line lower at 6 months compared to preoperative CDVA (*p* = 0.002), whereas this occurred in only 6.4% of eyes for the MEL90 (*p* = 0.003) (Figure 2). A total of 77.7% of eyes in the EX500 and 88.0% of eyes in the MEL90 achieved the same or better postoperative UDVA compared to their preoperative CDVA. However, eyes treated with EX500 were more likely to improve by at least one line of UDVA compared to baseline CDVA than those treated with MEL90 (41.6% vs. 16.1%, respectively; *p* < 0.001).

#### 3.2.2. Hyperopia with and Without Astigmatism

**Figure 3:**
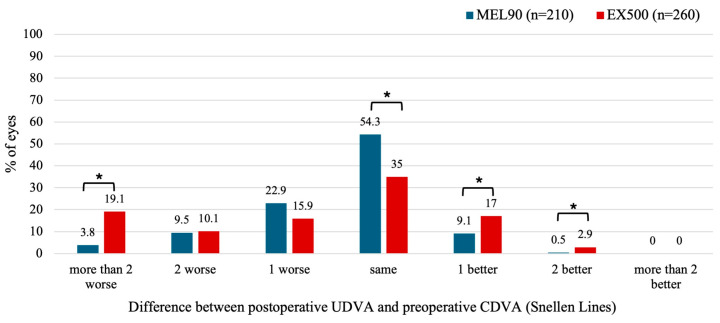
Preoperative corrected distance visual acuity (CDVA) versus uncorrected distance visual acuity (UDVA) for the hyperopic cohort at 6 months. LogMAR values from the MEL90 studies were converted to Snellen equivalents for comparison. * Indicates statistically significant (*p* < 0.05).

At 6 months, 19.1% of eyes treated with EX500 showed more than two lines of UDVA worse compared to baseline CDVA, whereas the MEL90 had only 3.8% (*p* < 0.001) (Figure 3). However, the EX500 was also more likely to improve postoperative UDVA by one (*p* = 0.013) or two lines (*p* = 0.047) relative to preoperative CDVA. Specifically, 19.9% of eyes in the EX500 group showed an improvement of at least 1 line of UDVA compared to the preoperative CDVA, whereas 9.6% of eyes in the MEL90 group did so (*p* < 0.05).

#### 3.2.3. Mixed Astigmatism

**Figure 4:**
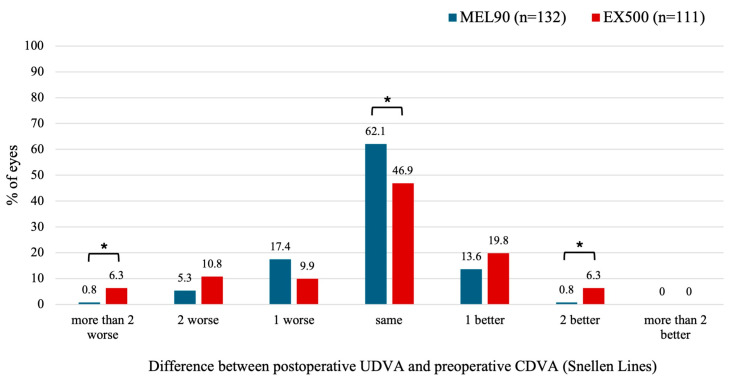
Preoperative corrected distance visual acuity (CDVA) versus uncorrected distance visual acuity (UDVA) for the mixed astigmatism cohort at 6 months. LogMAR values from the MEL90 studies were converted to Snellen equivalents for comparison. * Indicates statistically significant (*p* < 0.05).

Compared to the MEL90, 5.5% more eyes in the EX500 cohort had a postoperative UDVA that was more than two lines lower than baseline CDVA (*p* = 0.025) (Figure 4). The EX500 cohort was also more likely than the MEL90 to improve by two lines of UDVA relative to preoperative CDVA (*p* = 0.025).

### 3.3. Safety

In all FDA studies, safety was evaluated by the number of eyes that lost more than two lines of CDVA compared to baseline within 6 months. Both MEL90 and EX500 demonstrated comparable safety profiles (Table 3). In the MEL90 myopia cohort, one eye transiently lost CDVA ≥2 lines and was retreated before the 3-month follow-up. Conversely, six eyes treated with the EX500 experienced a loss of ≥2 lines of CDVA, though this difference was not statistically significant (*p* > 0.05). Although four eyes in the hyperopia cohort lost CDVA ≥2 lines with the EX500 compared to none with MEL90, this was not significant (*p* > 0.05).

### 3.4. Accuracy

**Figure 5:**
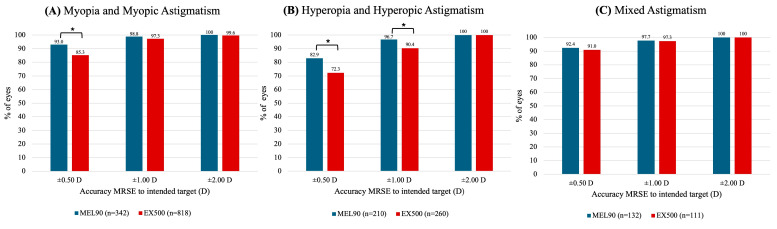
Accuracy of the manifest refraction spherical equivalent (MRSE) to the intended target for (**A**) myopia and myopic astigmatism, (**B**) hyperopia and hyperopic astigmatism, and (**C**) mixed astigmatism. * Indicates statistically significant (*p* < 0.05).

In the myopic cohort, more eyes treated with the MEL90 (93.0%) achieved an MRSE within ±0.50 D of the intended target at 6 months, compared to 85.3% of eyes in the EX500 group (*p* < 0.001) (Figure 5A). Both platforms in this cohort demonstrated that at least 97% of eyes were within ±1.00 D and ±2.00 D of target MRSE (both *p* > 0.05). For the hyperopic cohort, the MEL90 was more likely to achieve an MRSE within ±0.50 D and ±1.00 D (both *p* = 0.007) of target (Figure 5B). Both platforms treated for mixed astigmatism showed comparable outcomes within ±0.50 D, ±1.00 D, and ±2.00 D of MRSE target (all *p* > 0.05) (Figure 5C).

### 3.5. Astigmatic Correction

The FDA PMA studies reported varying preoperative cylinder values; therefore, only comparable refractive bins (up to 4.0 D of cylinder) were included in the analysis. The preoperative cylinder range of 0 to 0.5 D could not be compared between the mixed astigmatism cohorts. Follow-up durations also differed, with data available only at 3 months for EX500 myopia and mixed astigmatism cohorts and at 6 months for all other cohorts across both platforms.

**Figure 6:**
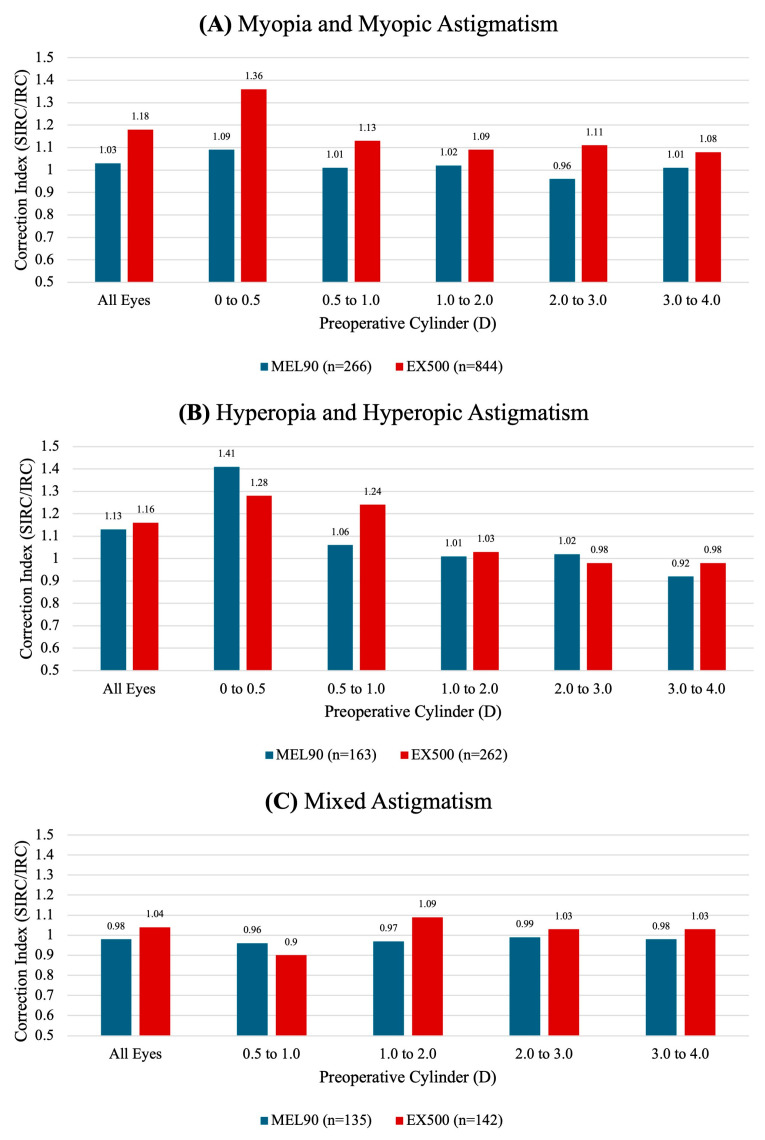
Correction indices of MEL90 and EX500 for (**A**) myopia and myopic astigmatism, (**B**) hyperopia and hyperopic astigmatism, and (**C**) mixed astigmatism stratified by preoperative cylinder.

In the myopic cohort, EX500 demonstrated a tendency for overcorrection at all preoperative refractive cylinders (Figure 6A). For eyes within 0.0 to 0.5 D of preoperative cylinder, the MEL90 showed a tendency to undercorrect in the myopia group and overcorrect in the hyperopia group relative to EX500 (Figure 6B). However, these differences could not be statistically compared.

### 3.6. Patient-Reported Outcomes

The PMA studies for the EX500 utilized a questionnaire with a graded scale (None-Mild, Moderate, or Marked-Severe) to assess subjective symptoms at baseline and 3 months postoperatively. In comparison, the MEL90 PMA studies implemented the Patient-Reported Outcomes with LASIK (PROWL) questionnaire to evaluate symptom resolution and development, both preoperatively and 6 months postoperatively.

Following EX500 in the myopic cohort, severe glare from bright lights decreased by 5.0%, and moderate glare decreased by 8.3%. Severe halos decreased by 4.1%, while moderate halos decreased by 7.5%. For MEL90, 74.2% (*n* = 66) of patients reported resolution of glare by 6 months postoperatively, while 6.5% (*n* = 108) reported the development of glare. For halos, 73.2% (*n* = 71) reported the resolution of halos, and 13.6% (*n* = 103) reported new symptoms.

In the hyperopic group, the EX500 platform showed a 7.8% reduction in severe glare and a 6.7% reduction in moderate glare by 3 months postoperatively. Severe and moderate halos decreased slightly by 0.5% and 0.3%, respectively. For MEL90, 82.6% (*n* = 46) reported resolution of glare, and 64.4% (*n* = 45) reported resolution of halos. Meanwhile, 10.9% (*n* = 64) developed new glare, and 24.6% (*n* = 65) reported new halos.

For mixed astigmatism, the EX500 cohort reported a 10.3% decrease in severe glare, but a 4.6% increase in moderate glare at 3 months postoperatively. Severe halos increased by 6.3%, while moderate halos decreased by 1.0%. Conversely, MEL90 outcomes showed that 82.1% (*n* = 28) of patients with preoperative glare reported resolution of symptoms at 6 months, while 8.3% (*n* = 48) reported new onset. Additionally, 58.3% (*n* = 36) reported resolution of halos, while 20.0% (*n* = 40) developed new symptoms.

### 3.7. Retreatment

For the EX500, 33 eyes in the myopic group, 16 eyes in the hyperopic group, and 3 eyes in the mixed astigmatism cohort were retreated. However, it should be noted that once an eye was retreated, they were excluded from subsequent reported outcomes. In comparison, the MEL90 PMA trials did not report outcomes on eyes that had been retreated.

At the 1-month follow-up after EX500 retreatment, 56.0% of eyes (14/25) in the myopic cohort achieved a UDVA of 20/20 or better. One eye (4.0%) lost CDVA ≥ 2 lines, while 64.0% of eyes (16/25) had MRSE within ±0.50 D of the intended target. For the hyperopic cohort, 54.6% of eyes (6/11) achieved a UDVA of 20/20 or better. No eyes (0/12) lost ≥ 2 lines of CDVA, and 83.3% of eyes (10/12) were within ±0.50 D of MRSE target. All eyes with mixed astigmatism achieved a UDVA of 20/20 or better and were within ±0.50 D of MRSE target.

### 3.8. Complications

Table 4 presents the intraoperative and postoperative complication rates for each cohort.

### 3.9. Review of Current Literature

After conducting a literature review, we selected four studies that reported visual outcomes on both the MEL90 and EX500 after treating myopia or myopic astigmatism with LASIK, to compare with the results from the PMA trial, as shown in Table 5. Due to the limited studies evaluating the MEL90 for hyperopia/hyperopic astigmatism and mixed astigmatism, all available studies were included. For a literature comparison with the EX500 PMA trial, we included one large retrospective cohort and one prospective cohort for treating hyperopia/hyperopic astigmatism, as well as two large retrospective cohorts for mixed astigmatism, as shown in Table 6 and Table 7, respectively.

## 4. Discussion

The MEL90 and EX500 platforms demonstrate comparable safety and efficacy outcomes that exceed the threshold recommended by the American National Standards Institute (ANSI) for excimer laser refractive surgery. According to the FDA and ANSI guidelines, an excimer laser must achieve at least 50% of eyes within ±0.50 D of MRSE target, and ≤5% of eyes losing more than two lines of CDVA [27,28,29]. Across all cohorts, fewer than 1.5% of eyes in the MEL90 and EX500 groups lost two or more lines of CDVA, surpassing the FDA benchmarks for safety (Table 3). The MEL90 achieved significantly greater MRSE accuracy within ±0.50 D of the target in both the myopia and hyperopia cohorts compared to EX500 (Figure 5). However, all cohorts in both platforms achieved at least 72.3% of eyes within ±0.50 D, exceeding the FDA threshold for MRSE accuracy.

It should be noted that the EX500 PMA trials included significantly older patients than the MEL90, with age differences of 5.0, 11.7, and 3.5 years for the myopic, hyperopic, and mixed astigmatism cohorts, respectively. This may partly explain the greater predictability observed in the MEL90 hyperopia cohort. In addition, while the EX500 is approved to treat myopia up to −12.0 D, which is higher than the −10.0 D for the MEL90, its preoperative MRSE was 0.68 D lower than the MEL90 cohort. In contrast, the proportion of eyes with a preoperative cylinder of 0.50 D or less was relatively similar between the two cohorts, at 46.3% for MEL90 and 50% for EX500.

Although comparative studies between the MEL series and the WaveLight series remain limited [30], retrospective analyses have evaluated the efficacy of each platform independently [16,19,21,23,24,25,31]. For the MEL90, Reinstein et al. reported that 92% of eyes with myopia and mixed astigmatism achieved a UDVA of 20/20 or better at 3 months without requiring nomogram adjustments [16], which exceeds the 86% reported in the PMA data. When treating for hyperopia with and without astigmatism, the authors reported a postoperative UDVA of 20/20 or better in 75% of eyes at one year postoperatively, which is greater than the 48.6% found in the PMA data at 6 months [21]. In the mixed astigmatism group, the authors demonstrated that 69% of eyes achieved a UDVA of 20/20 or better at 1 year, which is comparable to the 68.9% reported in the PMA trial at 6 months [24].

For the EX500, Niparugs et al. showed that 90% of eyes with myopia and myopic astigmatism achieved a 12-month postoperative UDVA of 20/20 or better, closely matching the 87.4% in the PMA study [19]. Durrie et al. observed superior hyperopia outcomes compared to the PMA data, with 84% of eyes reaching UDVA of 20/20 or better at 6 months, exceeding the 68.7% reported in the PMA trial [23]. Another study found excellent efficacy for the EX500 in the treatment of mixed astigmatism, with 74% of eyes achieving a UDVA of 20/20 or better at 12 months [25]. This exceeds the reported 69.4% of eyes achieving the same outcome in the EX500 PMA data at 6 months. Biscevic et al. also showed that the EX500 can successfully reduce astigmatism along the horizontal (J0) meridian in eyes with mixed and myopic astigmatism [31].

In this comparative study, MEL90 and EX500 achieved similar cylinder reduction. Published studies have demonstrated that MEL90 achieved a residual cylinder of ≤0.50 D in 90% to 97% of eyes in the myopic cohort, 75% in the hyperopic cohort, and 65% in the mixed astigmatism cohort (Table 5, Table 6 and Table 7). In comparison, the EX500 showed similarly predictable astigmatism in the myopic cohort (92% to 97%) and in the hyperopic cohort (76%), with a higher rate in the mixed astigmatism cohort (78.4% to 100%). Despite recent advancements and the use of refined nomograms, it is evident that the treatment of hyperopia lags behind that of myopia and mixed astigmatism. One contributing factor may be the large angle kappa, defined as the difference between the pupillary and visual axes, which can compromise centration and result in less optimal efficacy and astigmatic correction [22]. However, the WaveLight ALLEGRETTO laser system, with an increased pulse frequency from 400 to 500 Hz, has shown greater improvement in correcting hyperopia and hyperopic astigmatism up to 6.00 D [32].

Patients reported experiencing an overall reduction in both subjective glare and halos across all refractive cohorts in the EX500, except for the mixed astigmatism cohort, where moderate glare and severe halos increased by 4.6% and 6.3%, respectively. This could be attributed to the use of additional pulses by the EX500, which selectively target the midperiphery to account for tangential energy loss. As a result, there are smoother transition zones and fewer induced higher-order aberrations, potentially reducing subjective visual disturbances such as glare or halos [33]. In comparison, over 50% of patients treated with MEL90 consistently showed symptom resolution for glare and halos across all groups.

The present study has a few limitations. As discussed previously, the FDA PMA studies reported varying preoperative parameters and clinical outcomes, which limited our analysis. Specifically, the differences in preoperative age and MRSE should be considered when interpreting the results, as they may be a confounding variable in all comparisons, particularly in the hyperopic cohorts. All comparisons were also limited to matched data across both platforms for consistency, with the exception of correction indices stratified by preoperative cylinder. The EX500 PMA clinical trials reported 12-month postoperative data separately for each cohort, while the MEL90 PMA provided 12-month data for all cohorts combined. Data stratified by cohort for the MEL90 were only available at the 6-month time point. This limited our ability to perform a comparative analysis beyond 6 months across all parameters. Similarly, patient-reported outcomes for halos and glare were included in the study, as these were the only parameters consistently reported across both platforms. All other subjective outcomes were excluded due to inconsistency in reporting. Additionally, differences in questionnaire formats for patient-reported outcomes prevented statistical comparison, so results were reported descriptively instead. Nine standard graphs for reporting visual outcomes proposed by Reinstein, Waring, and Alpins could not be comprehensively replicated due to the lack of raw data and inconsistent data reporting across PMA trials [34,35]. Instead, available data were stratified by refractive error and plotted to show overall trends and help clinicians assess platform performance over time. Nevertheless, the strength of our analysis is supported by the large sample sizes across all cohorts, which included 1259 myopic, 511 hyperopic, and 297 mixed astigmatic eyes available for direct comparison. Future research should aim to address these limitations by using standardized patient questionnaires and including additional subjective parameters beyond halos and glare. Such studies would provide a more comprehensive evaluation of each platform’s clinical significance in terms of subjective visual symptoms.

## 5. Conclusions

This comparative analysis of the FDA PMA studies for the Zeiss MEL90 and the Alcon WaveLight EX500 illustrates the advancements in corneal refractive surgery technology and may aid clinicians in selecting the most appropriate device for refractive surgery. Both platforms exceeded the safety and efficacy benchmarks set by the FDA and ANSI in the treatment of myopia with and without astigmatism, hyperopia with and without astigmatism, and mixed astigmatism. Across all refractive cohorts, the EX500 was more likely to improve by one or two lines of UDVA from baseline, while the MEL90 more frequently maintained preoperative CDVA. However, the MEL90 demonstrated greater MRSE accuracy in both the myopic and hyperopic cohorts. No platform showed a clear advantage over the other in terms of astigmatic correction. Despite the limitations of this analysis, both excimer lasers demonstrate excellent visual outcomes in terms of safety, predictability, and efficacy, with low complication rates and performance exceeding the FDA benchmarks.

## Figures and Tables

**Table 1:** Zeiss and Alcon excimer laser specifications.

	Zeiss			Alcon	
Excimer Laser	MEL80	MEL90	Allegretto Wave	Allegretto Wave Eye-Q	EX500
Approval (year)	2006	2024	2003	2007	2010
Type	ArF	ArF	ArF	ArF	ArF
Wavelength (nm)	193	193	193	193	193
Frequency (Hz)	250	500	200	400	500
Eye Tracker (Hz)	250	1050	200	400	1050
Pulse Duration (ns)	4–7	4–7	10	10	6
Optical Zone (mm)	6.0–6.5	6.0–7.0	4.5–8.0	4.5–8.0	6.0–6.5
Ablation Zone (mm)	9.2 ^a^	9.2 ^a^	9.0	9.0	9.0
Peak Fluence (mJ/cm^2^)	>150	>150	400	400	400

Abbreviations: ArF = Argon Fluoride. ^a^ Has the ability to ablate up to 10.0 mm.

**Table 2:** Patient demographics.

	MEL90		EX500						
Parameter	Myopia ± Astigmatism	Hyperopia ± Astigmatism	Mixed Astigmatism	Myopia ± Astigmatism	Hyperopia ± Astigmatism	Mixed Astigmatism	*p* ^d^	*p* ^e^	*p* ^f^
Eyes (*n*)	358	221	135	901	290	162			
Sex, male/female (*n*)	88/95	52/65	46/31	436 ^b^/465 ^b^	142 ^b^/148 ^b^	109 ^b^/53 ^b^			
Age (years) ^a^	33.1 ± 7.5 (19 to 63)	39.9 ± 11.5 (18 to 62)	35.5 ± 9.1 (20 to 57)	38.07 ± 9.7 (18 to 67)	51.55 ± 8.8 (25 to 69)	39.0 ± 9.4 (22 to 70)	<0.001	<0.001	0.001
Preoperative MRSE (D) ^a^	−5.14 ± 3.26	2.48 ± 1.83	−0.04 ± 1.49	−4.46 ± 2.35 ^c^	2.27 ± 1.30 ^c^	−0.21 ± 0.05 ^c^	<0.001	0.156	0.192
Preoperative CYL (D) ^a^	−1.00 ± 1.05 (−4.00 to 0.00)	−0.99 ± 1.04 (−4.00 to 0.00)	−2.60 ± 0.99 (−4.00 to −0.75)	−0.85 ± 0.86 ^c^ (−5.00 to 0.00)	−0.66 ± 0.73 ^c^ (−4.50 to 0.00)	−2.40 ± 1.15 ^c^ (−6.00 to 0.00)	0.017	<0.001	0.110

Abbreviations: MRSE = manifest refraction spherical equivalent; D = diopters; CYL = cylinder. ^a^ Values expressed as mean ± standard deviation (range reported for age and preoperative cyl). ^b^ Represented by the number of eyes. ^c^ Calculated based on a weighted average. ^d^ *p*-value of myopia ± astigmatism cohort between MEL90 and EX500. ^e^ *p*-value of hyperopia ± astigmatism cohort between MEL90 and EX500. ^f^ *p*-value of mixed astigmatism cohort between MEL90 and EX500.

**Table 3:** Comparison of safety parameters from baseline to 6 months postoperative.

	MEL90			EX500					
Parameter (*n*/N) (%)	Myopia ± Astigmatism	Hyperopia ± Astigmatism	Mixed Astigmatism	Myopia ± Astigmatism	Hyperopia ± Astigmatism	Mixed Astigmatism	*p* ^c^	*p* ^d^	*p* ^e^
CDVA loss ≥ 2 lines	1/342 (0.5%)	0/210 (0%)	0/133 (0%)	6/818 (0.7%)	4/260 (1.5%)	1/111 (0.9%)	0.131	0.455	0.681
CDVA worse than 20/40 ^a^	0/342 (0%)	0/210 (0%)	0/133 (0%)	0/818 (0%)	1/260 (0.4%)	0/111 (0%)	NA	NA	NA
CDVA worse than 20/25 ^a^	1/342 (0.5%)	0/210 (0%)	0/133 (0%)	2/779 (0.3%)	0/241 (0%)	0/97 (0%)	0.998	NA	NA
Increased CYL > 2.0 D	0/342 (0%)	0/210 (0%)	1/133 (0.8%)	0/242 ^b^ (0%)	0/79 ^b^ (0%)	–	NA	NA	NA

Abbreviations: CDVA = corrected distance visual acuity; CYL = cylinder; D = diopters; NA = not applicable (no statistical test performed due to absence of events in both groups). – Indicates there is no data available. ^a^ If preoperative CDVA was 20/20 or better. ^b^ Eyes treated for spherical correction only. ^c^ *p*-value of myopia ± astigmatism cohort between MEL90 and EX500. ^d^ *p*-value of hyperopia ± astigmatism cohort between MEL90 and EX500. ^e^ *p*-value of mixed astigmatism cohort between MEL90 and EX500.

**Table 4:** Intraoperative and postoperative complications for the MEL90 and EX500 clinical trials.

	MEL90			EX500		
Complication *n* (%)	Myopia ± Astigmatism (*n* = 358)	Hyperopia ± Astigmatism (*n* = 221)	Mixed Astigmatism (*n* = 135)	Myopia ± Astigmatism (*n* = 876)	Hyperopia ± Astigmatism (*n* = 285)	Mixed Astigmatism (*n* = 161)
Interface debris ^a^	13 (3.6)	1 (0.5)	0	0	0	0
Persistent FBS/pain	0	1 (0.5)	0	7 (0.8)	5 (1.8)	2 (1.2)
Epithelium in interface	4 (1.1)	4 (1.8)	0	3 (0.3)	7 (2.5)	0
Retinal detachment	1 (0.3)	0	0	0	1 (0.4)	0
DLK	6 (1.7)	0	0	0	0	0
Corneal striae	4 (1.1)	0	0	0	0	0
Persistent corneal edema	1 (0.3)	2 (1.5)	2 (1.5)	0	0	0
Epithelial defect	1 (0.3)	0	0	16 (1.8)	3 (1.0)	0
Flap dislocation	1 (0.3)	0	0	2 (0.2)	0	0
Flap tear/damage	0	2 (0.9) ^a^	0	0	0	0

Abbreviations: FBS = foreign body sensation; DLK = diffuse lamellar keratitis. ^a^ Intraoperative complication.

**Table 5:** Comparison of visual outcomes for the treatment of myopia with and without astigmatism between MEL90 and EX500 in published studies.

Study (Year)	Country	Follow-Up (mo)	*N*	MRSE, Mean ± SD		CYL ≤0.5 D	MRSE ±0.5 D	MRSE ±1.0 D	UDVA ≥20/20	UDVA ≥20/40	Loss of ≥2 Lines of CDVA	Loss of 1 Line of CDVA	No Change of CDVA	**Gain of 1 Line** **of CDVA**	**Gain of** **≥** **2 Lines of CDVA**	**Safety** **Index**	**Efficacy** **Index**
				**Preop (D)**	**Postop (D)**												
**MEL90**																	
PMA trial (2024) [6]	US	6	342	−5.14 ± 3.26	-	-	93.0	98.8	86.3	98.8	0.5	3.2	71.9	22.2	2.4	-	-
Shehata et al. (2023) ^a^ [13]	EGY	6	150	−4.89 ± 0.77	−0.05 ± 0.05	-	98.7	-	54	-	-	-	-	-	-	-	-
Vaswani et al. (2021) [14]	UK	3	382	-	-		91	98	92	-	0	-	-	-	-	-	-
Brar et al. (2021) [15]	IND	12	165	−3.98 ± 1.90	−0.23 ± 0.23	97	91	100	96	100	0	3	59	35	3	1.08	1.00
Reinstein et al. (2015) [16]	UK	3	286	−3.83 ± 1.83	−0.13	90	88	100	92	99 ^b^	0	6	59	31	4	-	-
**EX500**																	
PMA trial (2003) [7]	US	12	901	−4.46 ± 2.35	-	-	85.1	97.7	87.4	99.0	0.5	-	-	-	-	-	-
Rowen et al. (2024) [17]	US	3	121	−4.35 ± 2.33	−0.01 ± 0.24	92	96	100	95	100	0	8	69	23	0	1.05 ^c^	0.98 ^d^
Agarwal et al. (2018) [18]	AUS	3	76	−2.49 ± 1.00	−0.09 ± 0.26	-	95	-	96.1	100	0	3	20	62	14	1.26 ^c^	1.12 ^d^
Niparugs et al. (2018) [19]	THA	12	254	−5.15 ± 2.41	−0.14 ± 0.30		91.3	98.5	89.0	98.7	0	14.0	58.8	27.2	0	-	-
Salés & Manche et al. (2013) [20]	US	12	34	−3.99 ± 1.71	−0.33 ± 0.34	97	85	97	97	100	0	15	47	32	6	-	-

Abbreviations: MRSE = manifest refraction spherical equivalent; SD = standard deviation; D = diopter; CYL = cylinder; Preop = preoperative; Postop = postoperative; UDVA = uncorrected distance visual acuity; CDVA = corrected distance visual acuity; Mo = months; US = United States; EGY = Egypt; UK = United Kingdom; IND = India; AUS = Australia; THA = Thailand. ^a^ Based on the weighted average from the low, medium, and high myopic cohorts. ^b^ Reported as % of eyes with UDVA of 20/30 or better. ^c^ Calculated using preoperative CDVA/postoperative UDVA. ^d^ Calculated using postoperative UDVA/preoperative CDVA.

**Table 6:** Comparison of visual outcomes for the treatment of hyperopia with and without astigmatism between MEL90 and EX500 in published studies.

Study (Year)	Country	Follow-Up (mo)	*N*	MRSE, Mean ± SD		CYL ≤0.5 D	MRSE ±0.5 D	MRSE ±1.0 D	UDVA ≥20/20	UDVA ≥20/40	Loss of ≥2 Lines of CDVA	Loss of 1 Line of CDVA	No Change of CDVA	**Gain of 1 Line** **of CDVA**	**Gain of** **≥** **2 Lines of CDVA**	**Safety** **Index**	**Efficacy** **Index**
				**Preop (D)**	**Postop (D)**												
**MEL90**																	
PMA trial (2024) [6]	US	12	210	2.48 ± 1.83	-	-	82.9	96.7	48.6	98.1	0	9.1	77.1	12.9	1.6	-	-
Reinstein et al. (2018) [21]	UK	12	1383	2.77 ± 1.34	−0.11 ± 0.55	75	73	93	75	99	0.6	17	64	19	0	-	-
**EX500**																	
PMA trial (2003) [8]	US	12	290	2.27 ± 1.30	-	-	65.3	90.8	67.5	98.8	1.5	-	-	-	-	-	-
Moshirfar et al. (2021) [22]	US	12	379	1.33 ± 1.10	−0.46 ± 0.79	76	78	96	69	97	1.1	4.8	74	19	1.1	1.03	0.93
Durrie et al. (2009) [23]	US	6	26	1.33 ± 0.76	0.16 ± 0.27	-	96.2	100	84	92	0	3.8	73.1	19.2	3.8	-	-

Abbreviations: MRSE = manifest refraction spherical equivalent; SD = standard deviation; D = diopter; CYL = cylinder; Preop = preoperative; Postop = postoperative; UDVA = uncorrected distance visual acuity; CDVA = corrected distance visual acuity; Mo = months; US = United States; UK = United Kingdom.

**Table 7:** Comparison of visual outcomes for the treatment of mixed astigmatism between MEL90 and EX500 in published studies.

Study (Year)	Country	Follow-Up (mo)	*N*	MRSE, Mean ± SD		CYL ≤0.5 D	MRSE ±0.5 D	MRSE ±1.0 D	UDVA ≥20/20	UDVA ≥20/40	Loss of ≥2 Lines of CDVA	Loss of 1 Line of CDVA	No Change of CDVA	Gain of 1 Line of CDVA	Gain of ≥2 Lines of CDVA	Safety Index	Efficacy Index
				**Preop (D)**	**Postop (D)**												
**MEL90**																	
PMA trial (2024) [6]	US	6	132	−0.04 ± 1.49	-	-	92.4	97.7	68.9	100	0	3.8	75.2	19.6	1.6	-	-
Reinstein et al. (2018) [24]	UK	12	105	−0.30 ± 0.90	−0.21 ± 0.38	65	85	99	73	94	0	10	57	32	1	-	-
**EX500**																	
PMA trial (2003) [9]	US	6	162	−0.98 ± 0.80	-	78.4	91.0	97.3	69.4	97.3	0.9	-	-	-	-	-	-
Moshirfar et al. (2022) [25]	US	12	179	−0.61 ± 0.70	−0.36 ± 0.57	80	88	100	74	100	0	3	72	25	0	1.02 ^a^	0.83 ^b^
Stonecipher et al. (2010) ^c^ [26]	US	6	111	0.78 ± 0.52	-	100	95	-	79	99	0	10	50	40	0	-	-

Abbreviations: MRSE = manifest refraction spherical equivalent; SD = standard deviation; D = diopter; CYL = cylinder; Preop = preoperative; Postop = postoperative; UDVA = uncorrected distance visual acuity; CDVA = corrected distance visual acuity; Mo = months; US = United States; UK = United Kingdom. ^a^ Calculated using preoperative CDVA/postoperative UDVA. ^b^ Calculated using postoperative UDVA/preoperative CDVA. ^c^ Data included for EX500 platform operating at 200 Hz pulse repetition rate.

## Data Availability

The original data presented in this study are openly available at the FDA database accessed at the following sites: https://www.accessdata.fda.gov/cdrh_docs/pdf6/P060004S006B.pdf, https://www.accessdata.fda.gov/cdrh_docs/pdf2/P020050.pdf, https://www.accessdata.fda.gov/cdrh_docs/pdf3/P030008B.pdf, https://www.accessdata.fda.gov/cdrh_docs/pdf3/P030008S004B.pdf, accessed on 3 May 2025, and in both the MEL90 and WaveLight EX500 Professional Use Information Booklets.

## Abbreviations

The following abbreviations are used in this manuscript:

LASIK	Laser assisted in situ keratomileusis
FDA	Food and Drug Administration
PMA	Premarket approval
SSED	Summary of Safety and Effectiveness Data
MRSE	Manifest refraction spherical equivalence
CDVA	Corrected distance visual acuity
UDVA	Uncorrected distance visual acuity
ArF	Argon Fluoride
CYL	Cylinder
D	Diopter
VA	Visual Acuity
PROWL	Patient reported outcomes with LASIK
FBS	Foreign body sensation
DLK	Diffuse lamellar keratitis
ANSI	American National Standards Institute
